# Differential cerebral white matter tract alterations in generalized anxiety disorder revealed by ultra-high-field 7 T diffusion-weighted imaging

**DOI:** 10.1016/j.jad.2026.121989

**Published:** 2026-05-20

**Authors:** Chang-Le Chen, Minjie Wu, Yueyang Chi, Max Rose, Jessica C. Weber, Shannon T. Lamb, Hecheng Jin, Tamer S. Ibrahim, Cecile D. Ladouceur, Yue-Fang Chang, Kuei Y. Tseng, Howard J. Aizenstein, Shaolin Yang

**Affiliations:** a Department of Psychiatry, University of Pittsburgh School of Medicine, Pittsburgh, PA, USA; b Department of Bioengineering, University of Pittsburgh, Pittsburgh, PA, USA; c Department of Neurosurgery, University of Pittsburgh, Pittsburgh, PA, USA; d Department of Anatomy and Cell Biology, College of Medicine, University of Illinois at Chicago, Chicago, IL, USA

**Keywords:** Generalized anxiety disorder, White matter, Correlational tractography, Diffusion tensor imaging, 7 T MRI

## Abstract

Generalized anxiety disorder (GAD) is characterized by chronic worry and emotional dysregulation, yet its underlying white matter (WM) microstructural alterations remain unclear owing to inconsistent findings and technical limitations in prior neuroimaging studies. To address this gap, we employed ultra-high-field 7-Tesla (7 T) diffusion tensor imaging (DTI) and advanced correlational tractography to characterize WM alterations in GAD, examine their associations with symptom burden, and evaluate their diagnostic utility. Eighty-eight young participants (27 with GAD and 61 healthy controls [HCs]) underwent 7 T DTI at 1.5-mm isotropic resolution. Whole-brain correlational tractography was applied to identify WM tracts exhibiting significant group differences in diffusion indices. Associations of identified WM tracts with Hamilton Anxiety Rating Scale (HAM-A) and Penn State Worry Questionnaire (PSWQ) scores were examined, and machine learning-based models were used to assess the diagnostic utility of the WM features. Two right-hemispheric tract alterations were identified in GAD. First, fractional anisotropy (FA) in the right cingulum was significantly elevated in GAD (*P_FDR_* < 0.001), particularly in the anterior portion of the tract. This elevated FA was positively correlated with HAM-A and PSWQ scores (*P_FDR_* < 0.001 for both), indicating that greater microstructural coherence was associated with more severe anxiety and worry. Second, lower FA was observed in the right prefrontal pathway (*P_FDR_* = 0.039), whereas this effect did not survive adjustment for medication use (*P_FDR_* = 0.133). Combining WM features with clinical scores improved diagnostic classification. These findings reveal tract-specific microstructural correlates of emotional dysregulation in GAD and provide new insight into its neurobiological mechanisms.

## 1. Introduction

Generalized anxiety disorder (GAD) is a highly prevalent and debilitating mental illness, with an estimated lifetime prevalence of 6.2% (Szuhany and Simon, 2022). It is characterized by chronic, excessive, and often uncontrollable worry that permeates multiple domains of a person's life, resulting in significant psychological distress and functional impairment (Yang et al., 2021). One prominent cognitive framework conceptualizes GAD as a disorder of emotion dysregulation (Goossen et al., 2019; Mochcovitch et al., 2014). Neuroimaging studies have implicated disrupted communication within the cortico-limbic circuitry, which integrates emotional processing with executive control (Duval et al., 2015; Kolesar et al., 2019). Functional magnetic resonance imaging (fMRI) studies have demonstrated reduced engagement of the prefrontal cortex (PFC) and anterior cingulate cortex (ACC) during emotional regulation tasks, findings commonly interpreted as impaired top-down control over limbic responses (Goossen et al., 2019; Kolesar et al., 2019; Makovac et al., 2016). However, the mechanisms underlying this cortical dysfunction remain incompletely understood. One hypothesis suggests that GAD is characterized by hypoactivity within the PFC/ACC, resulting in insufficient emotional regulation and heightened amygdala reactivity (Makovac et al., 2016; Qiao et al., 2017). In contrast, some fMRI studies have reported increased prefrontal activation during resting-state and task-based paradigms, suggesting an overactive top-down control system associated with chronic worry (Mochcovitch et al., 2014; Mohlman et al., 2017). Together, these abnormalities in functional connectivity (FC), particularly altered coupling between the amygdala and the PFC/ACC, are thought to play a central role in the core symptom of excessive worry.

Such functional disturbances may arise from structural alterations in white matter (WM) tracts that anatomically connect the PFC, ACC, and limbic regions (Fonzo and Etkin, 2017). Diffusion tensor imaging (DTI), which assesses WM microstructural integrity using metrics such as fractional anisotropy (FA), has been widely applied to examine structural alterations in GAD (Kolesar et al., 2019). Numerous DTI studies have reported reduced FA in key cortico-limbic pathways, suggesting compromised WM integrity (Tromp et al., 2012; Wang et al., 2016). These microstructural abnormalities are frequently observed in the uncinate fasciculus, as well as other tracts implicated in anxiety-related networks, including the corpus callosum, cingulum, anterior thalamic radiation, inferior fronto-occipital fasciculus, inferior longitudinal fasciculus, and corona radiata (Phan et al., 2009; Tromp et al., 2012; Tromp et al., 2019; Wang et al., 2019; Yang et al., 2020). However, findings regarding WM structural networks in GAD remain mixed in the literature, with some studies reporting focal increases in FA (Feng et al., 2019; Kolesar et al., 2019; Yang et al., 2020). This heterogeneity suggests a complex, regionally variable profile of WM alterations in GAD, which is also potentially influenced by methodological limitations that contribute to inconsistent findings across studies (Harrewijn et al., 2021; Kolesar et al., 2019).

Limitations of published neuroimaging studies include reliance on MRI scanners with lower magnetic field strengths (1.5 T or 3 T), which provide limited signal-to-noise ratio (SNR) and reduce the reliability of detecting subtle WM microstructural abnormalities. Additional challenges arise from the use of diverse DTI analytical approaches (e.g., region-based analysis or tract-based spatial statistics (Lu et al., 2018)) and high heterogeneity across study cohorts, driven by small and mixed samples, differences in comorbid conditions, medication status, and broad age ranges. Collectively, these confounding factors hinder the ability to isolate neurobiological alterations specific to GAD.

Here, we implemented DTI on an ultra-high-field (7 T) MRI scanner to investigate WM alterations in GAD. The 7 T MRI provides higher SNR, which is particularly important for diffusion-weighted imaging because it is inherently noisier than conventional structural imaging. Improved SNR may enhance the reliability of diffusion measures and increase sensitivity to subtle WM abnormalities (Choi et al., 2011). Prior work has also shown that 7 T imaging can improve statistical sensitivity and reduce the required sample size relative to 3 T (Chu et al., 2025). We also employed an advanced tractometry approach, correlational tractography, to delineate and examine potential alterations in WM tracts associated with GAD at the whole-brain level. This approach offers a more data-driven and anatomically specific assessment of WM, enabling a comprehensive and fine-grained detection of microstructural changes that may have been overlooked in prior studies. To further reduce confounding influences related to chronic illness, aging, and long-term medication exposure, we focused on a sample of young adults with GAD.

We hypothesized that individuals with GAD would exhibit specific structural alterations in diffusion-derived measures within WM tracts critical to cortico-limbic emotion regulation circuitry. We further examined whether these microstructural alterations were associated with the psychological burden of core symptoms, including worry and anxiety. Finally, we employed machine learning-based models to evaluate the clinical utility of these microstructural metrics, testing whether WM alterations could serve as objective markers to improve the diagnostic classification of GAD. The overall goal of the present study was to generate robust, high-resolution evidence to refine, and potentially reconcile, competing neurobiological models, thereby advancing efforts to identify reliable WM biomarkers for GAD.

## 2. Materials and methods

### 2.1. Participants

A total of 88 participants were recruited for this study through Pitt+Me, the participant recruitment portal of the University of Pittsburgh Clinical and Translational Science Institute. GAD diagnoses were established by clinical psychiatrists according to the Diagnostic and Statistical Manual of Mental Disorders, Fifth Edition (DSM-5), and confirmed by experienced clinical psychologists through a medical record review process. Exclusion criteria for all participants across the groups included: (1) current or past major neurological or systemic medical illness; (2) current or past schizophrenia, bipolar disorder, posttraumatic stress disorder, mania, intellectual disabilities, obsessive-compulsive disorder, or social phobia; (3) history of alcohol abuse or dependence within the last 12 months; (4) self-reported history of substance abuse within the last 2 months; (5) contraindications to MRI (e.g., metallic implants, claustrophobia, or pregnancy); (6) the Wechsler Abbreviated Scale of Intelligence (Second Edition) <70 or >130; and (7) moderate or severe traumatic brain injury. Additionally, GAD participants with current major depressive episode at enrollment or non-remitted major depressive disorder (MDD), treatment with high-dose psychotropic medications or any antipsychotics, or presenting a risk to themselves or others were also excluded. Detailed psychiatric comorbidity and medication information is provided in Supplementary Material S1. HCs were group-matched to the patient cohort by age, sex, and years of education. During the screening, research staff confirmed the absence of any current or past psychiatric disorders, and the exclusion criteria for all the participants across the groups were applied, with the additional requirement of no family history of psychiatric illness. The study protocol was approved by the Institutional Review Board (IRB) of the University of Pittsburgh and conducted in accordance with the Declaration of Helsinki. Written informed consent was obtained from all participants prior to enrollment. During clinical assessment, all participants completed the Hamilton Anxiety Rating Scale (HAM-A) and Penn State Worry Questionnaire (PSWQ) to quantify anxiety and worry severity.

### 2.2. MRI data acquisition and processing

Diffusion-weighted images were acquired on a 7 T human MRI scanner (Siemens Magnetom, Erlangen, Germany) with a custom-built 60Tx/32Rx Tic-Tac-Toe (TTT) head radiofrequency (RF) coil system (Sajewski et al., 2025; Santini et al., 2021). Specifically, we employed a single-shell DTI sequence with the following imaging parameters: repetition time (TR) = 6500 ms, echo time (TE) = 81.6 ms, number of b_0_ images = 4, diffusion sampling directions = 46, *b*-value = 1010 s/mm^2^, in-plane resolution = 1.5 mm, and slice thickness = 1.5 mm. This diffusion protocol was selected to balance data quality (spatial resolution, angular sampling, and SNR) and feasibility (tolerable scan duration for the study cohort) (Choi et al., 2011). Preprocessing of the diffusion-weighted imaging data began with a bias correction step to mitigate signal inhomogeneity (Tournier et al., 2019). Next, the susceptibility artifact was estimated using reversed phase-encoding b_0_ images processed with TOPUP in the TinyFSL package (https://fsl.fmrib.ox.ac.uk/fsl/). Subsequently, eddy current and head movement corrections were performed using FSL's EDDY, implemented through the integrated preprocessing interface in DSI Studio (Yeh, 2025). The accuracy of the b-table orientation was then validated by comparing fiber orientations against a population-averaged template (Yeh et al., 2018). Finally, the corrected DWI data were reconstructed in the standard Montreal Neurological Institute (MNI) space using *q*-space diffeomorphic reconstruction (QSDR) that nonlinearly transforms the images to the ICBM152 adult template (Yeh and Tseng, 2011). This process yielded the spin distribution function using a diffusion sampling length ratio of 1.25 and an isotropic output resolution of 1.5 mm (Yeh et al., 2010). The diffusion scalar indices, including FA and mean diffusivity (MD), were estimated for use in the connectometry analysis. Additionally, structural T1-weighted images were acquired using a 3D MPRAGE sequence (TR = 3000 ms; TE = 1.96 ms; inversion time = 1200 ms; flip angle = 8°; 0.75 mm isotropic resolution). Lateral ventricle volume was estimated from these images using the CAT12 toolbox (Gaser et al., 2024) and included as a covariate in the tractography analyses.

### 2.3. Correlational tractography

Diffusion MRI connectometry analysis was performed using correlational tractography to identify WM pathways in which local diffusion indices (i.e., FA and MD) were correlated with group differences (Yeh et al., 2016). A nonparametric Spearman partial correlation was used to assess voxel-wise associations between changes in diffusion indices along fiber pathways and group, while controlling for age, sex, years of education, handedness, and lateral ventricle volume using multiple regression. Controlling for lateral ventricle volume was intended to reduce the potential influence of partial volume effects at the ventricle-WM interface. The analysis included all 88 participants. A *t*-score threshold of 2.5 was applied (Salisbury et al., 2024; Zhang et al., 2025), and correlational tractography was conducted using a deterministic fiber tracking algorithm (Yeh et al., 2013). To evaluate the robustness of the findings, additional sensitivity analyses were performed using alternative *t*-score thresholds of 2.0, 3.0, and 3.5 (Supplementary Material S2). The cerebellum was excluded due to signal variation across subjects, and whole-brain seeding was implemented in the MNI space. The resulting tracts were refined using topology-informed pruning with 16 iterations (Yeh et al., 2019). To correct for multiple comparisons, a false discovery rate (FDR) threshold of 0.05 was applied, and statistical significance was determined by estimating the null distribution of track length through 4000 randomized permutations of the group labels. This procedure was applied identically and independently to the FA-based and MD-based correlational tractography analyses. The coordinates of the identified WM tract bundles were saved and subsequently used to extract local diffusion indices for downstream statistical analysis.

### 2.4. Clinical assessments

To assess anxiety severity and worry-related symptoms, all participants completed the HAM-A and PSWQ. The HAM-A (Hamilton, 1959) is a clinician-administered instrument designed to evaluate the severity of both psychic and somatic anxiety symptoms. It comprises 14 items, each rated on a 5-point scale from 0 (absent) to 4 (very severe), yielding total scores ranging from 0 to 56, with higher scores indicating greater anxiety severity. Scores of 0–17 indicate no-to-mild anxiety, 18–24 mild-to-moderate anxiety, 25–30 moderate-to-severe anxiety, and 31 and above severe-to-very-severe anxiety. The PSWQ (Meyer et al., 1990) is a self-report measure that assesses the tendency toward excessive and uncontrollable worry, a core feature of GAD. It comprises 16 items, each rated on a 5-point scale from 1 (not at all typical of me) to 5 (extremely typical of me), producing a total score ranging from 16 to 80, with higher scores reflecting greater levels of trait worry. We used the total scores from both the HAM-A and PSWQ to index overall anxiety severity and trait worry in the study cohort.

### 2.5. Statistical analysis

We first performed a confirmatory group comparison using analyses of covariance (ANCOVAs) adjusted for age, sex, years of education, handedness, and lateral ventricle volume. Multiple comparisons were corrected using the Benjamini-Hochberg method, accounting for the number of identified tract bundles. To evaluate the potential influence of antidepressant exposure, additional sensitivity analyses were performed controlling for fluoxetine-equivalent dose over the preceding three months (Supplementary Material S1). Because tract bundles comprised streamlines extending across multiple regions, we further examined spatial patterns of WM alterations by equally dividing the identified bundles into three main segments along their principal spatial orientations. Separate ANCOVAs were then conducted for each segment with the same covariates, and multiple comparison correction was applied to control for alpha inflation. Standardized effect sizes and statistical power were also reported for completeness and interpretability.

To examine clinical associations of altered WM bundles, we performed linear regression analyses with robust estimation to test whether diffusion indices of the identified tracts could explain variance in psychological burden as measured by total scores on the HAM-A and PSWQ while adjusting for the same covariates used in the ANCOVAs. Post hoc analyses were also conducted on individual tract segments to evaluate the spatial pattern of effect sizes using partial correlation, with Benjamini-Hochberg correction applied to control for multiple comparisons.

Finally, to assess the potential of the identified WM tracts as diagnostic-aided markers, we conducted a classification analysis to differentiate patients with GAD from HCs using both psychological scores and tract-based diffusion indices. HAM-A and PSWQ total scores were used as indicators of psychological burden in worry and anxiety. Five support vector machine (SVM) classifiers were trained (Noble, 2006): one for each questionnaire score (HAM-A and PSWQ), one combining the two scores, one using WM features alone, and one all-in-one model combining clinical scores and WM features. Before model training, all input features were adjusted for the effects of covariates. The SVM classifiers were implemented in MATLAB R2023a via the *fitcsvm* function, utilizing a *ν*-SVC formulation with a cubic polynomial kernel (*ν* = 0.5, with other default hyperparameters). This framework is optimal for binary classification in low-dimensional feature spaces (Chen et al., 2024). Model performance was evaluated via receiver operating characteristic (ROC) analysis, with accuracy, sensitivity, and specificity estimated through a leave-one-out bootstrap cross-validation procedure (Efron and Tibshirani, 1997). In practice, each of the 88 participants was sequentially held out, and 50 bootstrap repetitions of size 87 were drawn with replacement from the remaining 87 participants to fit independent SVM models, each predicting the held-out case. Performance metrics were computed across the held-out predictions, with means and 95% confidence intervals derived from the bootstrap distribution. This procedure combines the structural fairness of leave-one-out validation with bootstrap resampling, which captures the sampling variability that deterministic leave-one-out validation alone cannot provide.

## 3. Results

### 3.1. Characteristics of study groups

The basic demographic and clinical characteristics of the study groups are shown in Table 1. There were no statistical differences in terms of age (*P* = 0.800), sex (*P* = 0.731), education (*P* = 0.584), and handedness (*P* = 0.670). The patients with GAD showed significantly higher scores of HAM-A and PSWQ than the HCs (*P*s < 0.001).

### 3.2. Distinct alterations of WM tract bundles in GAD

**Fig. 1.**
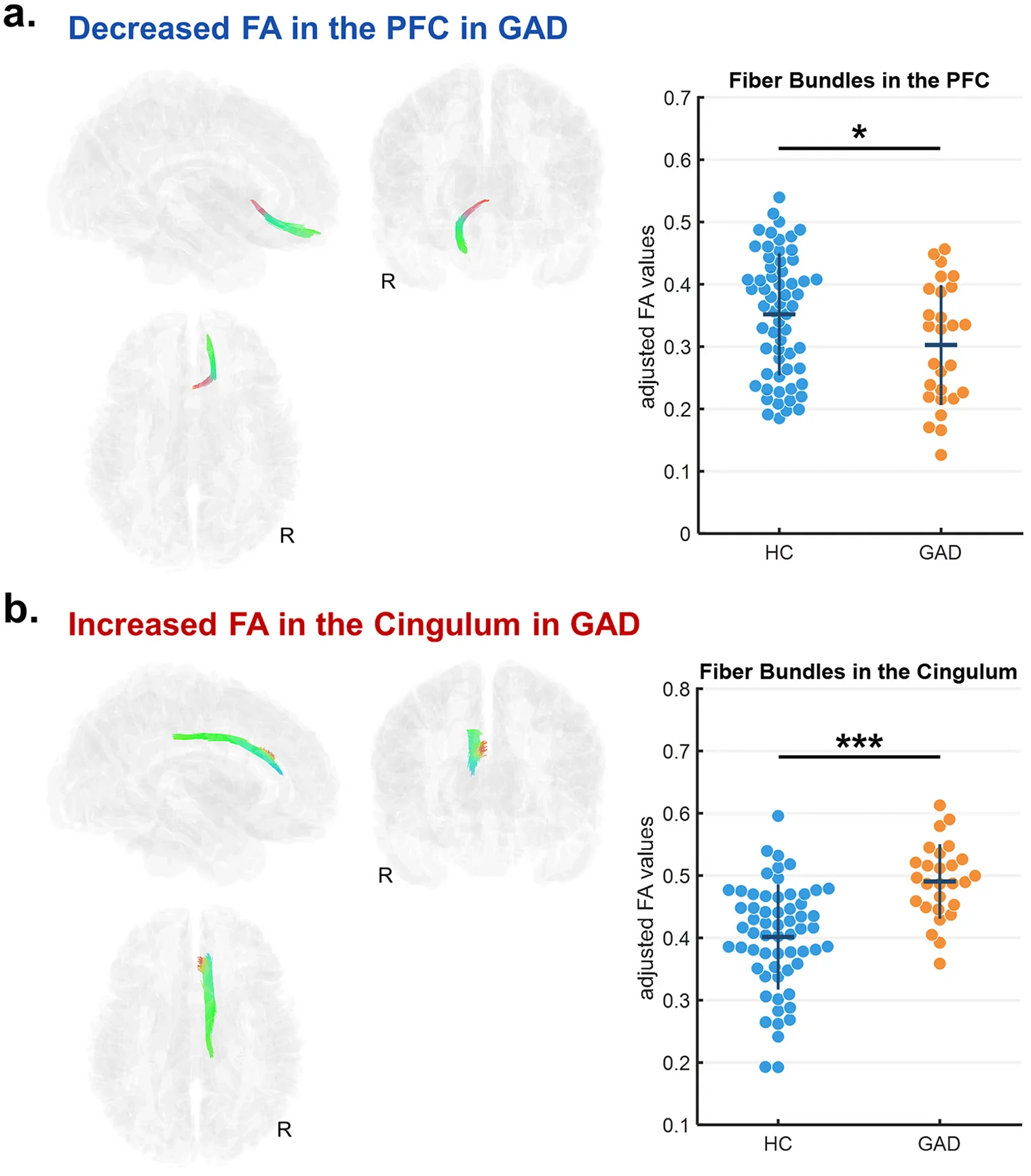
Correlational tractography of WM pathways associated with GAD based on FA. Two distinct patterns of altered connectivity were observed: (a) fiber bundles connecting the right limbic region to the right PFC showed significantly decreased FA in the patients with GAD, whereas (b) fiber bundles within the right cingulum demonstrated significantly increased FA in the patients with GAD, both compared with healthy control participants (HCs). Analyses controlled for age, sex, education, handedness, and lateral ventricle volume. The tract bundles are color-coded to indicate orientation; green, red, and blue represent the anterior-posterior, left-right, and superior-inferior directions, respectively. Abbreviation: FA, fractional anisotropy; GAD, generalized anxiety disorder; PFC, prefrontal cortex; WM, white matter.

Whole brain FDR-corrected correlational tractography identified two fiber tract bundles showing significantly altered FA associated with GAD. The confirmatory group comparison indicated that one tract bundle connecting the right limbic region to the right PFC exhibited significantly decreased FA in patients with GAD compared with the HCs (GAD: 0.303 ± 0.096, HCs: 0.352 ± 0.098, *F*_(1,81)_ = 4.40, *P_FDR_* = 0.039, partial *η*^2^ = 0.052, Cohen's *f* = 0.233, power = 0.580) (Fig. 1a). In contrast, another tract bundle within the right cingulum demonstrated significantly increased FA in patients with GAD relative to HCs (GAD: 0.491 ± 0.060, HCs: 0.401 ± 0.084, *F*_(1,81)_ = 22.74, *P_FDR_* < 0.001, partial *η*^2^ = 0.219, Cohen's *f* = 0.530, power = 0.998) (Fig. 1b). These two identified tract bundles were not significantly correlated with each other according to the partial correlation analysis (*r* = −0.119, *P* = 0.285). After controlling for fluoxetine-equivalent dose, the decreased FA in the right prefrontal pathway in patients with GAD became insignificant (GAD: 0.312 ± 0.095, HCs: 0.348 ± 0.098, *F*_(1,81)_ = 2.31, *P_FDR_* = 0.133, partial *η*^2^ = 0.028, Cohen's *f* = 0.170) while the increased FA in the right cingulum in GAD remained significant (GAD: 0.489 ± 0.059, HCs: 0.402 ± 0.084, *F*_(1,81)_ = 13.32, *P_FDR_* < 0.001, partial *η*^2^ = 0.143, Cohen's *f* = 0.409). Correlational tractography analysis based on MD did not identify any tract bundles surviving FDR correction at the same statistical threshold applied to the FA analysis. Sensitivity analyses using different *t*-score thresholds (2.0, 3.0, and 3.5) showed generally consistent findings, with expected differences in tract extent across thresholds. The results at *t* = 2.5 were the most stable and interpretable and were further supported by the confirmatory group comparisons (Supplementary Material S2).

**Fig. 2.**
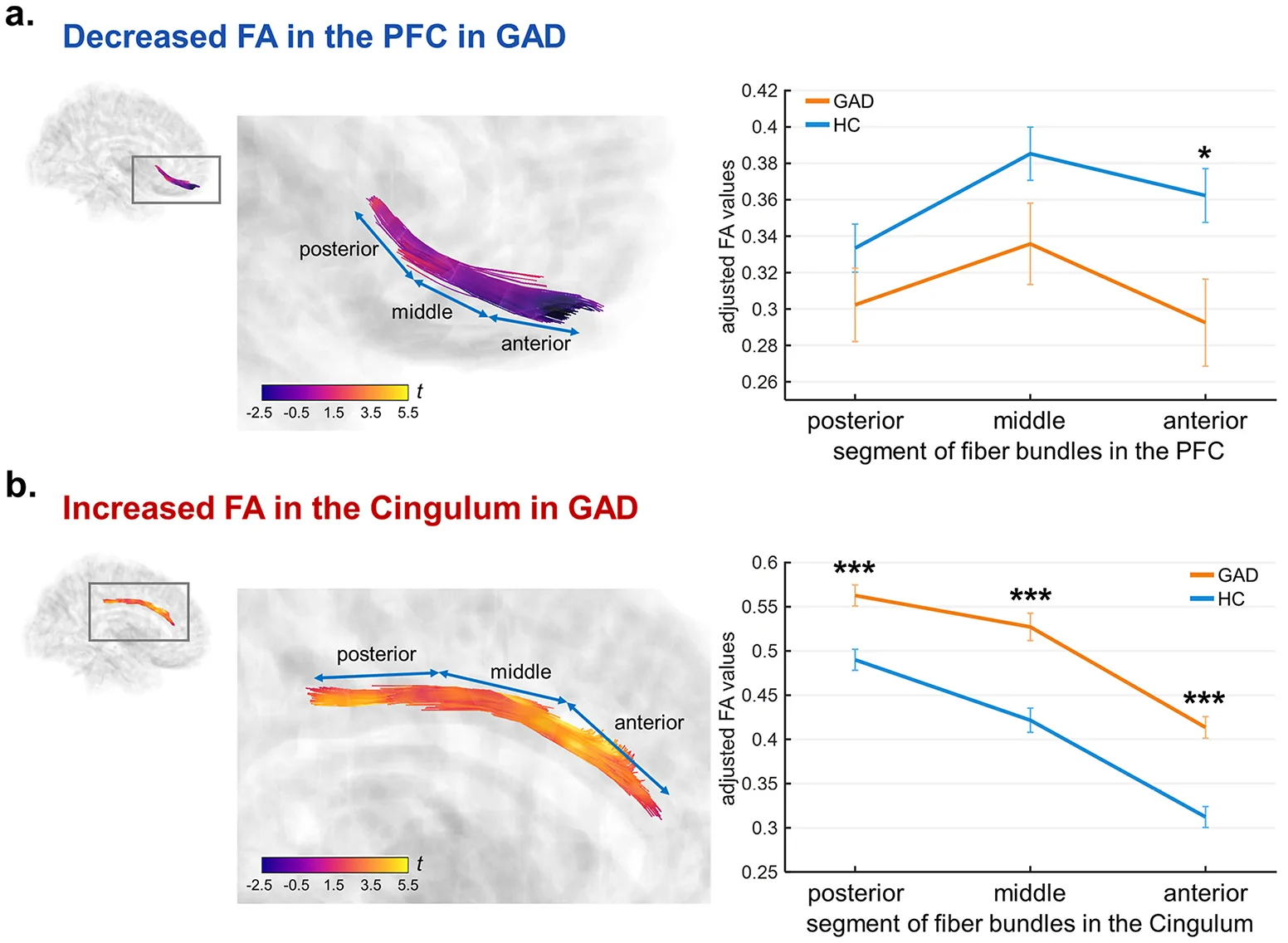
Segment-wise group comparisons of FA along the identified WM tracts. Each tract was divided into anterior, middle, and posterior segments along the anterior–posterior axis. (a) The tract connecting the right limbic region to the right PFC showed a significant FA reduction in patients with GAD compared with HCs, primarily in the anterior segment. (b) The right cingulum tract exhibited significantly higher FA in GAD, compared with HCs, across all segments, with the effect most pronounced in the anterior (frontal) region. Analyses controlled for age, sex, education, handedness, and lateral ventricle volume. The tract bundles are color-coded to represent the corresponding statistical *t*-values. Abbreviation: FA, fractional anisotropy; GAD, generalized anxiety disorder; WM, white matter.

For the tract bundles showing significantly altered FA in GAD, we further conducted segment-wise group comparisons along their principal orientations. As both identified bundles were association fibers extending along the anterior-posterior axis, each fiber bundle was divided into anterior, middle, and posterior segments. In the tract connecting to the right PFC, the posterior and middle segments showed no significant group differences (posterior region, GAD: 0.302 ± 0.105, HCs: 0.333 ± 0.103, *F*_(1,81)_ = 1.56, *P_FDR_* = 0.215, partial *η*^2^ = 0.019, Cohen's *f* = 0.139; middle region, GAD: 0.336 ± 0.116, HCs: 0.385 ± 0.114, *F*_(1,81)_ = 3.23, *P_FDR_* = 0.114, partial *η*^2^ = 0.038, Cohen's *f* = 0.200) whereas the anterior segment demonstrated a significant reduction in FA in patients with GAD compared with HCs (anterior region, GAD: 0.293 ± 0.124, HCs: 0.362 ± 0.115, *F*_(1,81)_ = 6.05, *P_FDR_* = 0.048, partial *η*^2^ = 0.070, Cohen's *f* = 0.273) (Fig. 2a). The effect size increased progressively from the posterior to the anterior segments, suggesting that FA reduction was more pronounced toward the prefrontal region in GAD. In contrast, the tract within the right cingulum exhibited significant group differences across all segments (posterior region, GAD: 0.563 ± 0.062, HCs: 0.490 ± 0.093, *F*_(1,81)_ = 12.79, *P_FDR_* < 0.001, partial *η*^2^ = 0.136, Cohen's *f* = 0.397; middle region, GAD: 0.527 ± 0.081, HCs: 0.422 ± 0.107, *F*_(1,81)_ = 19.40, *P_FDR_* < 0.001, partial *η*^2^ = 0.193, Cohen's *f* = 0.489; anterior region, GAD: 0.413 ± 0.063, HCs: 0.312 ± 0.092, *F*_(1,81)_ = 24.82, *P_FDR_* < 0.001, partial *η*^2^ = 0.235, Cohen's *f* = 0.554), with GAD patients showing higher FA values, particularly in the middle and anterior portions of the tract (Fig. 2b). Similarly, effect sizes increased toward the anterior (frontal) region, indicating a gradient of more prominent FA elevation in the frontal segments of the right cingulum.

### 3.3. Clinical associations of identified WM tract bundles with psychological burden in worry and anxiety

**Fig. 3.**
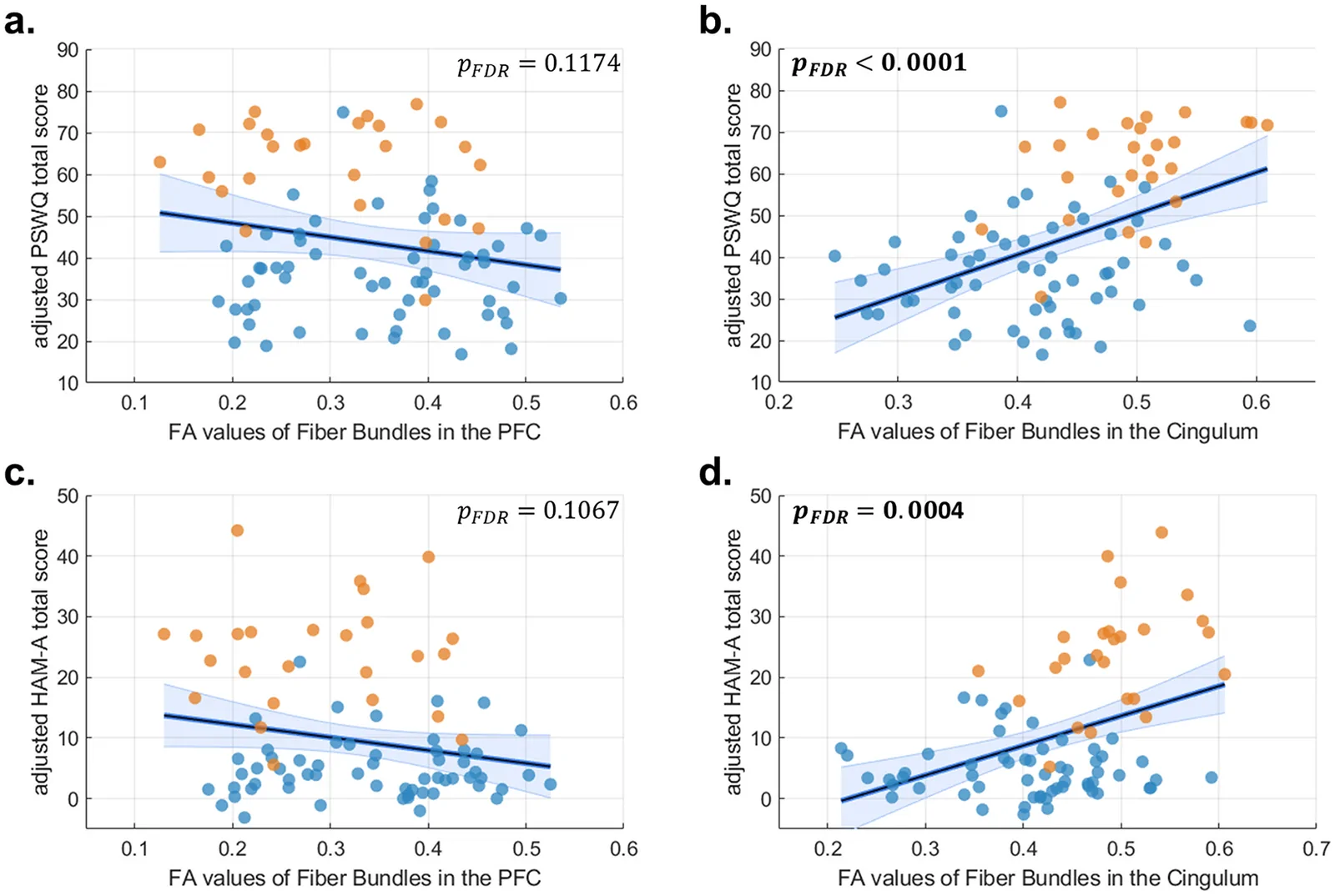
Associations between FA in the identified WM tracts and psychological burden in worry and anxiety. Panels (a) and (c) show that FA values of the tract bundle in the right PFC were not significantly correlated with PSWQ or HAM-A score. Panels (b) and (d) illustrate that FA values in the right cingulum tract showed significant positive associations with both PSWQ and HAM-A scores, indicating that higher FA values were linked to greater trait worry and general anxiety. Analyses controlled for age, sex, education, handedness, and lateral ventricle volume. Orange and blue colors indicate patients with GAD and HCs, respectively. *P* values are displayed to four decimal places. Abbreviation: FA, fractional anisotropy; GAD, generalized anxiety disorder; HAM-A, Hamilton Anxiety Rating Scale; HC, healthy control; PFC, prefrontal cortex; PSWQ, Penn State Worry Questionnaire.

We investigated whether the identified tract bundles were associated with psychological burden in worry and anxiety, as measured by the HAM-A for general anxiety and the PSWQ for trait worry, across the combined GAD and HC samples. Regression analyses showed that FA values in the prefrontal tract bundle were not significantly correlated with either PSWQ or HAM-A scores (PSWQ, β = −32.5, standard error [SE] = 20.5, 95% confidence interval [C.I.] = [−73.3, 8.4], *P_FDR_* = 0.117, partial *η*^2^ = 0.031, Cohen's *f*^2^ = 0.032, power = 0.386; HAM-A, β = −21.6, SE = 12.2, 95% C.I. = [−45.8, 2.6], *P_FDR_* = 0.107, partial *η*^2^ = 0.042, Cohen's *f*^2^ = 0.044, power = 0.490) (Fig. 3a and c). In contrast, FA values in the right cingulum tract exhibited significant positive associations with both measures (PSWQ, β = 98.1, SE = 21.7, 95% C.I. = [54.9, 141.4], *P_FDR_* < 0.001, partial *η*^2^ = 0.216, Cohen's *f*^2^ = 0.275, power = 0.998; HAM-A, β = 49.1, SE = 12.6, 95% C.I. = [23.9, 74.2], *P_FDR_* < 0.001, partial *η*^2^ = 0.158, Cohen's *f*^2^ = 0.188, power = 0.980) (Fig. 3b and d), indicating that higher FA values were linked to greater burden in trait worry and general anxiety. Within the GAD group alone (n = 27), partial correlations between right cingulum FA and clinical scores did not reach statistical significance after FDR correction (PSWQ, β = 76.3, SE = 40.7, 95% C.I. = [−9.0, 161.6], *P_FDR_* = 0.153, partial *η*^2^ = 0.178, Cohen's *f*^2^ = 0.216, power = 0.632; HAM-A, β = 50.6, SE = 35.3, 95% C.I. = [−23.5, 124.7], *P_FDR_* = 0.168, partial *η*^2^ = 0.093, Cohen's *f*^2^ = 0.103, power = 0.355).

**Fig. 4.**
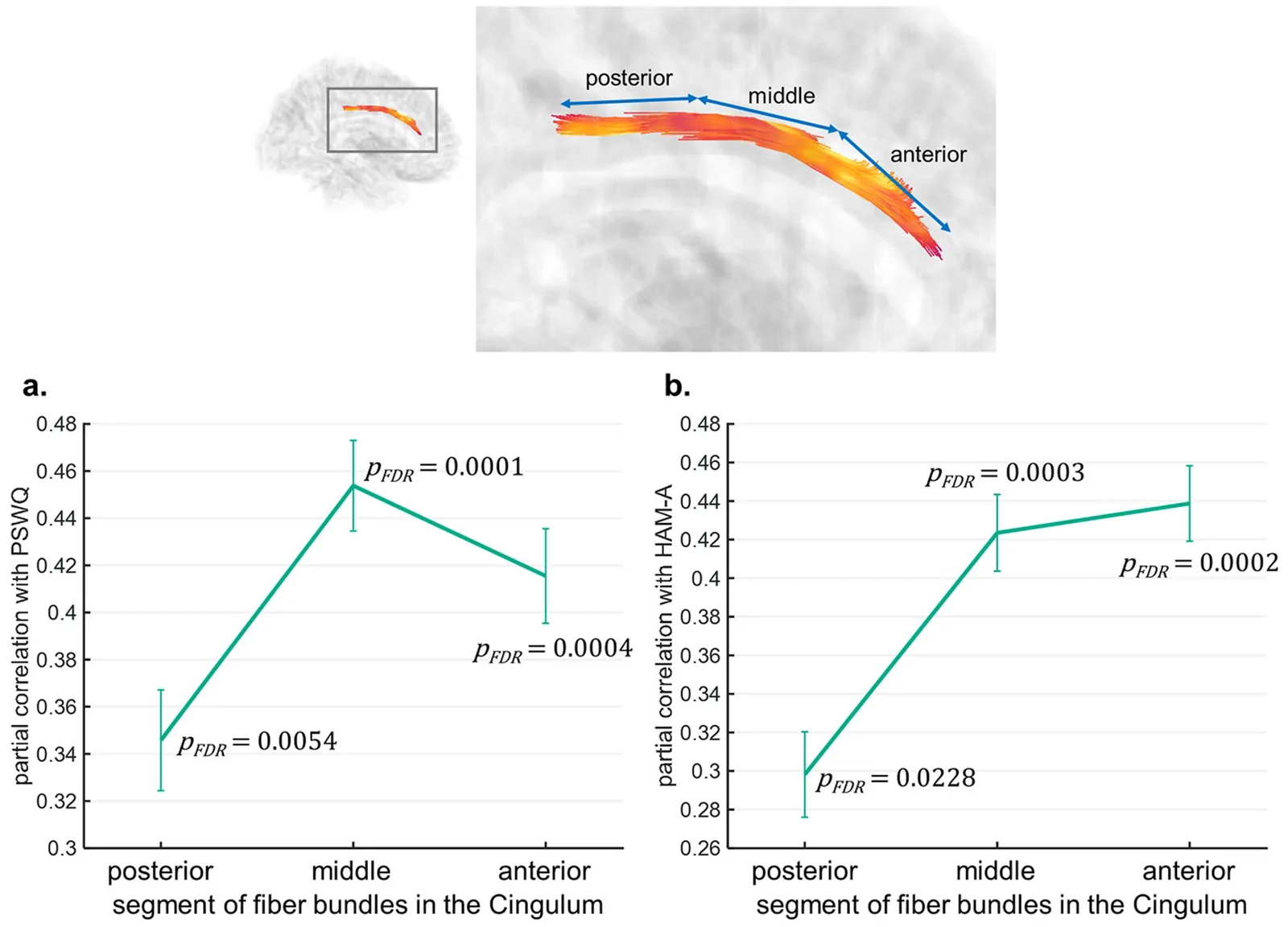
Segment-wise associations between FA in the right cingulum and psychological burden in worry and anxiety. Panels (a) and (b) show the partial correlation coefficients of PSWQ and HAM-A scores with FA values across segments, respectively. Analyses controlled for age, sex, education, handedness, and lateral ventricle volume. Error bars represent 95% confidence intervals of the estimates. *P* values are displayed to four decimal places. Abbreviation: FA, fractional anisotropy; HAM-A, Hamilton Anxiety Rating Scale; PSWQ, Penn State Worry Questionnaire.

Similarly, we examined the segment-wise associations of the right cingulum tract with worry and anxiety in the pooled sample. For PSWQ, the middle segment showed the strongest partial correlation (*rho* = 0.454, 95% C.I. = [0.435, 0.473], *P_FDR_* < 0.001), followed by the anterior (*rho* = 0.416, 95% C.I. = [0.395, 0.436], *P_FDR_* < 0.001) and posterior (*rho* = 0.346, 95% C.I. = [0.324, 0.367], *P_FDR_* = 0.005) segments (Fig. 4a). For HAM-A, the anterior segment exhibited the greatest positive correlation (*rho* = 0.439, 95% C.I. = [0.419, 0.458], *P_FDR_* < 0.001), followed by the middle (*rho* = 0.424, 95% C.I. = [0.404, 0.443], *P_FDR_* < 0.001) and posterior (*rho* = 0.298, 95% C.I. = [0.276, 0.320], *P_FDR_* = 0.023) segments (Fig. 4b). These findings align with our earlier observation that the anterior portion of the right cingulum (close to the frontal region) shows a stronger group effect of GAD compared with the more posterior segments.

### 3.4. Improvement in classification of GAD versus HC using identified WM tract bundles

**Fig. 5.**
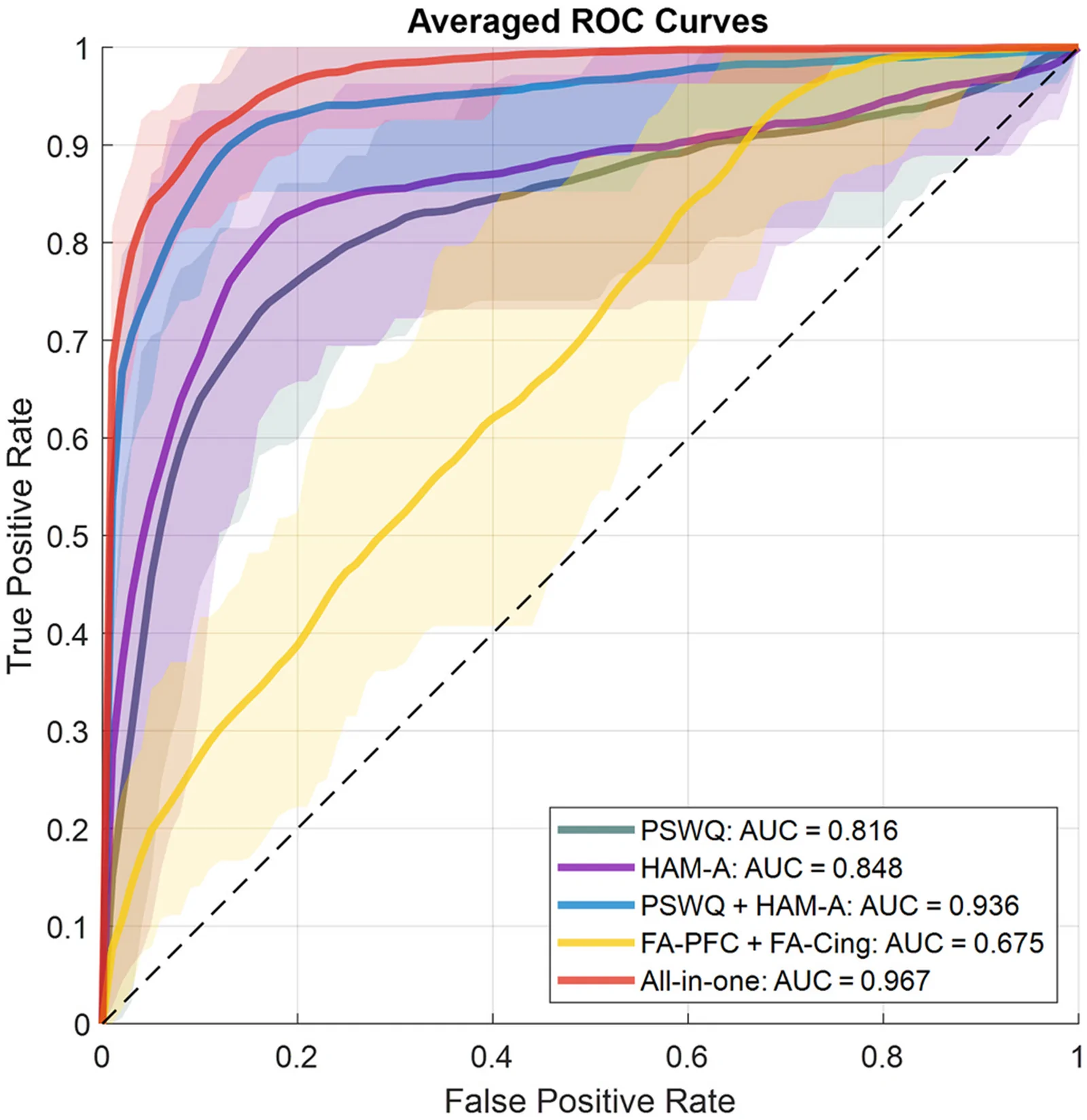
ROC analysis for GAD classification based on clinical scores and identified WM tract features. Average ROC curves with 95% confidence bands were generated using different combinations of clinical scores and/or WM features, and classification performance was evaluated using leave-one-out bootstrap cross-validation with 50 repetitions. Abbreviation: AUC, area under the curve; Cing, cingulum; FA, fractional anisotropy; HAM-A, Hamilton Anxiety Rating Scale; PFC, prefrontal cortex; PSWQ, Penn State Worry Questionnaire; ROC, receiver operating characteristic.

To evaluate the clinical utility of the identified WM tract bundles as diagnostic aided markers, we conducted a classification analysis to differentiate the GAD and HC groups using different combinations of clinical scores (i.e., PSWQ and HAM-A) and WM features. Using SVM modeling with a cubic kernel and leave-one-out bootstrap cross-validation repeated 50 times, the baseline models incorporating PSWQ averagely achieved an accuracy of 81.5% (95% C.I. = [74.7%, 87.8%]) and an area under the ROC curve (AUC) of 0.816 (95% C.I. = [0.733, 0.878]) (Fig. 5). The corresponding average sensitivity and specificity were 0.647 (95% C.I. = [0.481, 0.787]) and 0.889 (95% C.I. = [0.811, 0.967]), respectively. Using HAM-A, the model achieved an accuracy of 83.7% (95% C.I. = [75.6%, 91.5%]) and an AUC of 0.848 (95% C.I. = [0.718, 0.949]). The corresponding sensitivity and specificity were 0.714 (95% C.I. = [0.556, 0.870]) and 0.892 (95% C.I. = [0.802, 0.951]), respectively. When combining both clinical scores (PSWQ and HAM-A), classification performance was enhanced, achieving an accuracy of 88.0% (95% C.I. = [84.9%, 91.2%]), an AUC of 0.936 (95% C.I. = [0.881, 0.971]), a sensitivity of 0.780 (95% C.I. = [0.694, 0.898]), and a specificity of 0.924 (95% C.I. = [0.877, 0.955]). Classification performance using only WM features (i.e., FA values from the right prefrontal and cingulum tracts) was relatively lower than that achieved with clinical measures, with an accuracy of 67.8% (95% C.I. = [62.2%, 73.3%]), an AUC of 0.675 (95% C.I. = [0.573, 0.744]), a sensitivity of 0.362 (95% C.I. = [0.213, 0.481]), and a specificity of 0.817 (95% C.I. = [0.738, 0.885]). However, overall classification performance was further improved by incorporating WM features into the all-in-one model (including all clinical measures and WM features) (Fig. 5). The final model achieved an accuracy of 91.1% (95% C.I. = [88.4%, 94.3%]) and an AUC of 0.967 (95% C.I. = [0.944, 0.981]), with high sensitivity (0.816 with 95% C.I. = [0.759, 0.889]) and high specificity (0.953 with 95% C.I. = [0.914, 0.984]). These results suggest that the identified WM tract features could provide additional information that enhances the differentiation between GAD and HCs. The coordinates of the identified tracts are available from the corresponding author upon request.

## 4. Discussion

The present study employed ultra-high-field 7 T DTI and correlational tractography to delineate fine-grained WM alterations in individuals with GAD compared with HCs. Two distinct, spatially dissociable WM tract abnormalities were identified: (1) decreased FA in fibers connecting the right limbic region and the right PFC, and (2) increased FA in the right cingulum. Notably, the most prominent alterations were observed in the anterior portions of both tracts, implicating prefrontal-limbic circuitry as a central structural substrate underlying anxiety pathophysiology. Furthermore, FA in the right cingulum was positively correlated with both anxiety and worry severity. These findings provide structural evidence supporting distinct alterations in fronto-limbic connectivity within models of emotion dysregulation in GAD.

The right cingulum exhibited increased FA in GAD compared with HCs, most prominently in its anterior and middle segments. These increases were positively correlated with both worry and anxiety scores. Elevated FA has been reported in several anxiety-related conditions (Zhang et al., 2011; Zhang et al., 2013). The cingulum is a major associative tract interlinking the medial PFC, parietal cortex, and hippocampal regions, which are core components of the default mode and limbic networks that support self-referential thought and emotion regulation (Kolesar et al., 2019; Mochcovitch et al., 2014). Increased FA of this tract may reflect altered microstructural organization arising from reduced axonal branching, decreased crossing fibers, reduced fiber dispersion, or changes in myelination (Figley et al., 2022). Anterior cingulate regions show aberrant neural activation during threat anticipation and the regulation of emotional conflict in GAD (Duval et al., 2015; Kolesar et al., 2019; Mochcovitch et al., 2014). This interpretation is further supported by our segment-wise correlation analyses, demonstrating that FA in the anterior cingulum was more strongly associated with worry- and anxiety-related burden. However, the within-GAD association analysis did not reach statistical significance, likely reflecting limited power to detect small-to-moderate effects rather than evidence of no association. Nonetheless, this finding cautions against interpreting the combined-sample results as reflecting continuous symptom-severity variation independent of diagnostic status.

In contrast, our finding of reduced FA in the prefrontal tract aligns with evidence implicating compromised structural integrity along fronto-limbic pathways in GAD (Tromp et al., 2012; Wang et al., 2016). The uncinate fasciculus, one major fronto-limbic pathway, has shown decreased FA in both adult and adolescent GAD cohorts (Tromp et al., 2012; Tromp et al., 2019), consistent with its role in mediating top-down regulation of limbic responses and facilitating cognitive control of affective states (Chen et al., 2019; Phan et al., 2009). Our finding of reduced FA toward the anterior segment suggests that diminished integrity of prefrontal terminations may specifically weaken regulatory input to subcortical emotion-processing regions (Chen et al., 2022; Mohlman et al., 2017; Phan et al., 2009). However, the reduction in FA within the prefrontal tract was insignificant after controlling for fluoxetine-equivalent dose, suggesting that this effect may be partially attributable to antidepressant exposure. Decreased FA in the prefrontal region was not significantly associated with worry or anxiety; nevertheless, the observed trend toward a negative correlation suggests a potential link between reduced WM integrity and greater psychological burden in worry and anxiety. These findings support a model in which weakened prefrontal WM connectivity compromises top-down control, potentially contributing to exaggerated limbic reactivity and sustained worry (Kolesar et al., 2019; Mohlman et al., 2017).

The absence of significant MD findings may reflect differences in the biological sensitivity of FA and MD. Sensitivity analyses across alternative *t*-score thresholds confirmed this pattern: at *t* = 2.0, a single subthreshold MD candidate (i.e., the left parietal cingulum) was identified but did not survive confirmatory testing, while at more stringent thresholds (*t* = 3.0 and *t* = 3.5), no MD tracts were identified (Supplementary Material S2). Prior work suggests that FA is often more sensitive to white matter organization, coherence, and myelination, whereas MD is more influenced by overall water mobility and extracellular diffusion (Alexander et al., 2007; Uddin et al., 2019). In relatively young cohorts such as the present sample, subtle tract-specific abnormalities may be more readily detected by FA than by MD (Lebel et al., 2012; Weinstein et al., 2011). This interpretation is also consistent with a previous DTI study of GAD, which reported group differences in FA, but not MD, in the uncinate fasciculus (Tromp et al., 2012). By contrast, MD-related changes may be more evident in aging or neurodegenerative conditions (Cox et al., 2016; Tseng et al., 2021). In addition, conventional MD measures may be susceptible to free-water and partial-volume effects, which could also reduce sensitivity to localized abnormalities (Chad et al., 2018; Pasternak et al., 2018).

In the present study, we demonstrated the capability of 7 T diffusion imaging combined with correlational tractography to resolve fine-scale WM alterations in GAD. In contrast to traditional predefined region- or tract-based approaches (Tung et al., 2021), our whole-brain tractometry method captured spatially continuous variations in diffusion metrics, revealing their along-tract gradients of biological effects. This analytic framework may help reconcile prior inconsistencies in the DTI literature, where findings often varied with how ROIs were selected. However, the tractography focused only on localized WM differences and does not capture large-scale network organization. Future work incorporating structural connectivity, functional connectivity, and structural–functional coupling, which has recently been shown to index both clinical pathology and individual variation in cognitive and emotional functioning beyond either modality alone (Suo et al., 2026; Suo et al., 2025), may provide a more comprehensive characterization of GAD-related brain alterations. Extending the multimodal framework to GAD could clarify whether the cingulum FA elevation observed in this study may translate into measurable disruptions within the default mode and salience networks anchored by the cingulum. The translational implications of the findings should be interpreted cautiously. Although 7 T diffusion MRI may improve sensitivity to subtle WM abnormalities, its clinical utility is currently limited by availability, cost, and technical complexity. Thus, the present results primarily advance the neurobiological understanding of GAD rather than provide an immediately applicable clinical biomarker. Future studies should test whether these findings can be replicated in larger cohorts and translated to more widely available platforms such as 3 T MRI. The integration of WM features into the machine-learning classifier further underscores their potential for clinical translation (Chiang et al., 2024). While clinical measures alone yielded relatively high diagnostic accuracy, incorporating diffusion MRI-derived indices improved classification performance beyond behavioral data, suggesting that structural connectivity metrics may serve as complementary biomarkers for individualized assessment of anxiety severity and treatment response.

Several limitations merit consideration. First, the relatively modest sample size, particularly in the GAD group, should be considered when interpreting the present results. Although the groups were well matched demographically, the limited sample size may reduce sensitivity to smaller effects and affect the stability and generalizability of the observed findings. Replication in larger, independent cohorts will be important to confirm the robustness of these results and to further establish their relevance to the neurobiology of GAD. Second, the cross-sectional design precludes causal inference and limits characterization of the temporal evolution of WM changes. Longitudinal studies are needed to clarify whether the observed microstructural differences reflect premorbid vulnerability, compensatory adaptation, or chronic sequelae of anxiety. Third, although the sample was restricted to a young age range and was imbalanced across study groups, the risk of GAD onset increases substantially during this developmental period. Future studies with broader age ranges and larger, more balanced samples are needed to capture a more complete trajectory of WM changes during the sensitive developmental period of anxiety-related disorders. Fourth, reliance on FA, a sensitive but nonspecific measure of microstructural integrity, should be complemented by additional diffusion metrics, such as neurite density and free-water imaging, as well as by multimodal integration of functional and metabolic data to refine mechanistic interpretations (Goossen et al., 2019).

In brief, this 7 T DTI study reveals a differential pattern of WM alterations in GAD, characterized by increased FA in the right cingulum and reduced FA in the right prefrontal pathway, both exhibiting an anterior predominance. These opposing microstructural changes likely reflect distinct facets of the disorder but collectively provide a structural framework for understanding the persistent worry and emotional dysregulation that are central to GAD. By integrating ultra-high-field tractometry with symptom-dimensional analysis, our findings help refine the current neurobiological model of GAD and underscore the potential of WM metrics as imaging markers for early detection and treatment stratification in anxiety disorders.

## Supplementary Material

1

## Figures and Tables

**Table 1:** Characteristics of the participants in the study.

Characteristics	GAD	HC	Comparisons
N	27	61	–
Age, year	21.9 (2.5)	21.7 (2.4)	*P* = 0.800
Sex, female, N (%)	20 (74.1%)	43 (70.5%)	*P* = 0.731
Education, year	15.5 (2.0)	15.2 (2.0)	*P* = 0.584
Handedness, right, N (%)	24 (88.9%)	50 (82.0%)	*P* = 0.670
PSWQ total score	62.7 (12.5)	35.9 (11.6)	*P* < 0.001
HAM-A total score	24.3 (9.2)	4.7 (4.8)	*P* < 0.001

Note: Mean and standard deviation are presented for continuous variables. Preliminary demographic comparisons between groups were conducted using two-sample *t*-tests for continuous variables and chi-square tests for categorical variables. Abbreviation: GAD, generalized anxiety disorder; HC, healthy control; PSWQ, Penn State Worry Questionnaire; HAM-A, Hamilton Anxiety Rating Scale.

## Appendix A. Supplementary data

Supplementary data to this article can be found online at https://doi.org/10.1016/j.jad.2026.121989.
