# Supplementary material for: Differential cerebral white matter tract alterations in generalized anxiety disorder revealed by ultra-high-field 7 T diffusion-weighted imaging
Source: J Affect Disord. Author manuscript; Available in PMC 2026 Sep 10. (PMC13561388; doi:10.1016/j.jad.2026.121989)
Supplement: Supplementary file 1 [file NIHMS2197647-supplement-1.docx]

**Supplementary Materials**

S1 Psychiatric comorbidity and medication status of the GAD group

S2 Sensitivity analysis of correlational tractography across different *t*-score thresholds

**S1 Psychiatric comorbidity and medication status of the GAD group**

Among the 27 participants with generalized anxiety disorder (GAD), 12 (44.4%) had no additional psychiatric comorbidities, whereas 15 (55.6%) had at least one comorbid psychiatric diagnosis. Depressive disorders were the most common comorbidity, present in 11 participants; notably, these cases reflected lifetime remitted major depressive disorder (MDD) or comorbid depressive disorders rather than current episodes. Attention-deficit/hyperactivity disorder (ADHD) was identified in 3 participants, and 1 participant had comorbid panic disorder. No participant met criteria for a current major depressive episode at enrollment, and none were taking antipsychotic medication, consistent with the study’s exclusion criteria. At the time of MRI, 10 of the 27 participants were taking antidepressants for GAD. Of these, 9 were taking selective serotonin reuptake inhibitors (SSRIs), and one was taking serotonin–norepinephrine reuptake inhibitors (SNRIs). No participants were receiving antipsychotic medication or high-dose psychotropic treatment. Antidepressant exposure was summarized as fluoxetine-equivalent dose (FEQ) using the well-established conversion framework (Hayasaka et al., 2015). To account for potential effects of antidepressant exposure, the average FEQ (mg/day) over the preceding three months was derived from medication history and included as a covariate in additional sensitivity analyses.

References:

Hayasaka, Y., Purgato, M., Magni, L.R., Ogawa, Y., Takeshima, N., Cipriani, A., Barbui, C., Leucht, S., Furukawa, T.A., 2015. Dose equivalents of antidepressants: Evidence-based recommendations from randomized controlled trials. J. Affect. Disord. 180, 179-184. https://doi.org/10.1016/j.jad.2015.03.021.

**S2 Sensitivity analysis of correlational tractography across different *t*-score thresholds**

To assess the robustness of the correlational tractography results, we repeated the primary analyses using *t*-score thresholds of 2.0, 3.0, and 3.5, while keeping all other analytical parameters identical to those used in the primary analysis (*t* = 2.5): FDR threshold = 0.05, permutations = 4,000, whole-brain seeding, topology-informed pruning with 16 iterations, and covariates including age, sex, years of education, handedness, and lateral ventricle volume. Confirmatory ANCOVA group comparisons (adjusted for the same covariates) were conducted on tracts identified at each threshold to verify the statistical significance of diffusion index (i.e., FA and MD) differences between the GAD and HC groups.

At *t* = 2.0, a broader set of candidate tracts were identified by the tractography. For decreased FA, the tract bundles in the left inferior fronto-occipital fasciculus (IFOF), left parahippocampal cingulum, combined corpus callosum forceps minor, and right corticostriatal tract showed lower FA in the GAD group than in healthy controls (Supplementary Figure 1 and Supplementary Table 1); however, none survived confirmatory group comparison after FDR correction. The tract bundles connected to the prefrontal cortex in the primary analysis appeared to involve portions of the combined corpus callosum forceps minor and right corticostriatal tract. For increased FA, the right frontal cingulum showed a robust group difference, with higher FA in the GAD group than in controls (Supplementary Figure 1 and Supplementary Table 1). Additional tracts showing increased FA at this threshold included the corpus callosum tapetum, right arcuate fasciculus, left frontal cingulum, and left arcuate fasciculus (Supplementary Figure 1 and Supplementary Table 1); however, these did not remain significant after FDR correction. For MD, only the left parietal cingulum showed a candidate decrease in the GAD group; however, this finding was not significant in confirmatory testing (Supplementary Figure 1 and Supplementary Table 1). At *t* = 3.0, the findings became more spatially restricted; only the right frontal cingulum remained identified, again showing significantly increased FA in the GAD group relative to controls (Supplementary Figure 1 and Supplementary Table 1). These identified tract bundles were consistent with those observed in the primary analysis at *t* = 2.5. At the more stringent threshold of *t* = 3.5, no significant tracts were identified.

This sensitivity analysis showed that varying the *t*-score threshold primarily affected the spatial extent and number of candidate tracts, whereas increased FA in the right frontal cingulum remained the most consistent and robust finding across thresholds. This pattern further supports the stability of the primary result obtained at *t* = 2.5.

**Supplementary Table 1**

***t = 2.0***

| **Decrease in FA** | **Tracts** | | **GAD** | **HC** | ***F*-values** | ***P_FDR_*-values** |
| --- | --- | --- | --- | --- | --- | --- |
|  | IFOF L | | 0.423 (0.046) | 0.448 (0.056) | 3.85 | 0.1062 |
|  | Cing ParaHip L | | 0.247 (0.025) | 0.256 (0.030) | 1.80 | 0.2096 |
|  | CC1+CST R | | 0.362 (0.106) | 0.385 (0.108) | 0.77 | 0.3815 |
| **Increase in FA** | **Tracts** | | **GAD** | **HC** | ***F*-values** | ***P_FDR_*-values** |
|  | Cing Front R | | 0.509 (0.063) | 0.424 (0.086) | 19.63 | < 0.0001 |
|  | CC2 | | 0.728 (0.044) | 0.688 (0.074) | 6.29 | 0.0564 |
|  | AF R | | 0.537 (0.041) | 0.516 (0.040) | 4.98 | 0.0757 |
|  | Cing Front L | | 0.547 (0.071) | 0.516 (0.070) | 3.20 | 0.1235 |
|  | AF L | | 0.502 (0.035) | 0.488 (0.038) | 2.65 | 0.1431 |
| **Decrease in MD** | | **Tracts** | **GAD** | **HC** | ***F*-values** | ***P*-values** |
|  | | Cing Parie L | 1.157 (0.151) | 1.208 (0.135) | 2.29 | 0.1344 |

***t = 3.0***

| **Increase in FA** | **Tracts** | **GAD** | **HC** | ***F*-values** | ***P*-values** |
| --- | --- | --- | --- | --- | --- |
|  | Cing Front R | 0.516 (0.069) | 0.416 (0.089) | 25.07 | < 0.0001 |

***t = 3.5***

**No significant tracts**

Abbreviation: AF, arcuate fasciculus; CC1, corpus callosum forceps minor; CC2, corpus callosum tapetum; Cing, cingulum; CST, corticostriatal tract; Front, frontal; IFOF, inferior fronto-occipital fasciculus; L, left; ParaHip, parahippocampal; Parie, parietal; R, right.

**Supplementary Figure 1. Visualization of identified tract bundles using correlational tractography at different *t*-score thresholds**
